# Diesel Exhaust Particles Remodel Lipid Raft-Associated Molecular Features Potentially Relevant to SARS-CoV-2 Susceptibility in A549 Cells

**DOI:** 10.3390/toxics14070642

**Published:** 2026-07-22

**Authors:** Laura Botto, Mario Mauri, Simone Serrao, Alessandra Bulbarelli, Elena Lonati, Emanuela Cazzaniga, Edoardo Ratti, Giuseppe Paglia, Paola Palestini

**Affiliations:** 1 School of Medicine and Surgery, University of Milano-Bicocca, 20900 Monza, Italy; laura.botto@unimib.it (L.B.); mario.mauri@unimib.it (M.M.); simone.serrao@unimib.it (S.S.); alessandra.bulbarelli@unimib.it (A.B.); elena.lonati1@unimib.it (E.L.); emanuela.cazzaniga@unimib.it (E.C.); e.ratti9@campus.unimib.it (E.R.); giuseppe.paglia@unimib.it (G.P.); 2 POLARIS Research Centre, University of Milano-Bicocca, 20900 Monza, Italy

**Keywords:** diesel exhaust particles (DEP), lipid rafts, ACE2, ADAM17, gangliosides, COVID-19

## Abstract

The overlap between the geographic distribution of COVID-19 outbreaks and pollution levels suggested a strong correlation between exposure to atmospheric particulate matter and an increased risk of developing severe forms of disease. This correlation has been highlighted by several epidemiological studies, indicating the existence of shared molecular mechanisms. Emerging evidence has highlighted the important role of lipid rafts in facilitating viral entry into cells. Specifically, the receptor binding domain of the SARS-CoV-2 spike protein interacts with sialylated glycans of the monosialic ganglioside GM1 and GM2 that are particularly enriched in lipid rafts. This interaction has been proposed to facilitate ACE2 recognition by the spike protein and may contribute to early events involved in viral attachment and entry. Here, we reveal that A549 alveolar lung cells, after DEP exposure, exhibit a significant shift in ACE2 into lipid rafts, accompanied by an increase in the immature form of ADAM17, the sheddase responsible for ACE2 cleavage. Additionally, DEP exposure results in a significant increase in IL-6 release, while no changes were observed in IL-8 and sACE2 release. This treatment does not cause significant alterations in protein levels or membrane redistribution of COX-2 and HO-1, proteins involved in the inflammatory response and oxidative stress following exposure to air pollution, and linked to COVID-19 pathogenesis. Finally, lipidomic analysis by UHPLC-MS revealed that DEP exposure induces a significant increase in GM2 levels, and a concomitant decrease in GM1 and GM3 levels. Together, these results indicate that DEP exposure remodels lipid raft-associated molecular features in A549 cells, including ACE2 membrane redistribution, altered ganglioside composition, and increased IL-6 release. Although these changes may be relevant to cellular mechanisms associated with SARS-CoV-2 susceptibility, the present study does not directly assess viral binding, viral entry, or infection, and further functional studies are required.

## 1. Introduction

Air pollution represents a critical environmental and public health challenge worldwide. Among airborne pollutants, ultrafine particles (UFPs; diameter < 100 nm) are of particular concern due to their ability to penetrate deep into the respiratory tract and translocate into the bloodstream and other organs [1,2]. Once internalized, UFPs induce oxidative stress, inflammatory responses, and immune dysregulation, all of which contribute to systemic toxicity and chronic disease development [3,4,5]. The COVID-19 (Coronavirus Disease 2019) pandemic has underscored the complex interplay between environmental exposure and infectious disease outcomes. Several epidemiological studies have revealed an overlap between regions with elevated air pollution levels and high COVID-19 incidence or mortality, suggesting that chronic exposure to airborne pollutants may exacerbate viral susceptibility and disease severity [6,7,8,9].

The renin-angiotensin system (RAS) has been identified as a crucial mediator of these interactions. The ACE2/Ang (1–7)/MAS axis exerts anti-inflammatory and protective effects, whereas the ACE/Ang II/AT1 axis promotes pro-inflammatory signaling [10]. Notably, ACE2, the cellular receptor exploited by SARS-CoV-2 for cellular entry, undergoes to proteolytic shedding by the metalloprotease ADAM17, a process implicated both in infection and in pollutant-induced inflammation [11].

Moreover, airborne particles have been shown to modulate the expression of inflammatory and oxidative stress markers such as cyclooxygenase-2 (COX-2) and heme oxygenase-1 (HO-1). In vivo studies demonstrated that acute exposure to biomass or diesel particles upregulates COX-2 levels in multiple organs [4,5]. Conversely, HO-1 exerts a protective role by catalyzing heme degradation into carbon monoxide (CO), biliverdin, and ferrous iron (Fe^2+^), thereby attenuating oxidative damage and inflammation [12]. Similarly, exposure to diesel exhaust particles (DEP) or biomass has been associated with increased HO-1 expression in several tissues [4,5].

Cytokines, including interleukin-6 (IL-6) and interleukin-8 (IL-8), are also upregulated in response to particulate exposure, amplifying the inflammatory response [13]. These same mediators are central to the immunopathology of SARS-CoV-2, where occurs an excessive cytokine release that lead to the so-called “cytokine storm” [14]. Elevated COX-2 expression has been correlated with higher mortality in COVID-19 patients [15], while HO-1 appears to mitigate infection-induced oxidative stress and cytokine-driven inflammation [16].

Recent in vivo mouse studies [17,18], have investigated whether exposure to PM2.5 or UFPs could affect the expression of ACE2 and ACE, revealing organ-specific changes in the ACE/ACE2 system that may affect SARS-CoV-2 pathogenesis. Uraki et al. [19], further implicates lipid rafts, specific membrane domains enriched in cholesterol and glycosphingolipid, as key determinants of SARS-CoV-2 entry. Gangliosides GM1 and GM2 within these domains interact with the viral spike protein, enhancing its binding to ACE2 and facilitating viral internalization. Since lipid rafts serve as dynamic signaling platforms, alterations in their composition may profoundly influence viral infectivity and host inflammatory responses.

Given that diesel engines constitute the primary source of UFPs in urban regions such as Lombardy (northern Italy), we investigated how exposure to diesel exhaust particles (DEP) affects lipid rafts composition and the expression of key proteins involved in SARS-CoV-2 infection and inflammation. Using human alveolar epithelial A549 cells as an in vitro model [20,21,22,23,24], we examined whether short-term sub-toxic DEP exposure, 1.6 µg/mL DEP [25], modifies the membrane localization of ACE2, ADAM17, COX-2, and HO-1, especially in lipid rafts, as well as the soluble ACE2 (sACE2) and pro-inflammatory cytokines IL-6 and IL-8 release.

At last, considering that structure defines function and subtle structural differences are closely linked to variations in cellular function, a lipid domain analysis was performed.

Understanding how DEP exposure alters membrane microdomain structure and inflammatory signaling may provide useful mechanistic insight into cellular changes potentially relevant to respiratory viral susceptibility. However, whether these DEP-induced molecular alterations translate into increased SARS-CoV-2 binding, entry, or infection requires dedicated functional validation.

## 2. Materials and Methods

### 2.1. Materials

All commercial chemicals were of the highest purity available and were purchased from Sigma Chemical Co., Ltd. (Milan, Italy). All cell culture stock solutions were supplied by Euroclone (Celbio, Milan, Italy). Precision Plus protein standards (All Blue) were purchased from Bio-Rad (Milan, Italy). The complete protease inhibitor cocktail was provided by Roche Diagnostics S.p.A. (Milan, Italy). The nitrocellulose membrane was from Amersham (GE Healthcare Europe GmbH, Milan, Italy).

The primary anti-Na/K-ATPase antibody was produced by Millipore (Massachusetts, USA); the anti-GM1, anti-ACE2 for enhanced chemiluminescence detection (ECL) and anti-ADAM17 were produced by Abcam (Cambridge, UK); anti-ACE2 for immunofluorescence staining was produced by GeneTex (Irvine, CA, USA); anti-HO-1 was produced by Cell Signaling Technology (Danvers, MA, USA); anti-COX2 was produced by BD Transduction Laboratories (Franklin Lakes, NJ, USA). Alexa Fluor 488-conjugated secondary antibodies, Alexa Fluor 594-conjugated Cholerae toxin B subunit (CTB), goat anti-mouse and/or anti-rabbit-HRP conjugates secondary antibodies for ECL detection and ECL SuperSignal detection kit were produced by Thermo Fisher Scientific (Waltham, MA, USA). Human IL-6 (high sensitivity) ELISA kit ENZ-KIT178-0001 and Human IL-8 (high sensitivity) ELISA kit ADI-900-156A were provided by Enzo Life Sciences (Farmingdale, NY, USA); Human ACE2 ELISA kit OKCD07767 was provided by AVIVA Systems Biology (San Diego, CA, USA). UHPLC solutions (LC-MS grade—LiChrosolv^®^) were provided by Merck KGaA (Darmstadt, Germany).

### 2.2. Cell Culture

Basal alveolar epithelial cells of human adenocarcinoma A549 (American Type Culture Collection, ATCC, Rockville, MD, USA) were used as an experimental model. The cells were seeded in Petri dishes, at a concentration of 175,000 cells per ml, in DMEM (Dulbecco’s Modified Eagles Medium) complete medium containing 10% fetal bovine serum, 1% penicillin and streptomycin, 1% glutamine, and maintained at 37 °C in a humidified atmosphere of 5% CO_2_. Cells were cultured for 24 h before exposure to DEPs, to allow adherence to the tissue culture plate.

### 2.3. DEP Treatment

Cells were treated with DEP SRM1650b (Standard Reference Material, National Institute of Standards and Technology, USA), a spent diesel particulate with a mean diameter of 0.18 μm, used in the literature as a model for ultrafine particulate matter [26].

Initially, 8 mg of DEP was resuspended in 5 mL of complete medium supplemented with 0.1% Tween-20, the detergent required to dissolve DEP, to give a concentration of 1.6 mg/mL. The resulting mixture was sonicated for 5 min on ice using an ultrasonic disruptor. The mixture was then diluted to 1.6 µg/mL in a complete medium for cell treatment [25].

The cells were then treated with DEP for 48 h. In parallel, control cells (CTR) were incubated with a complete DMEM medium, while control Tween cells (CTR-T) were incubated with a complete DMEM medium supplemented with Tween-20 at the same concentration as in the DEP treatment (0.0001%).

The DEP concentration and exposure time used in this study were selected as a short-term, sub-toxic in vitro stimulation condition based on previous work and confirmed by cell viability analysis. This experimental setting was not intended to reproduce chronic human exposure directly, but rather to investigate early molecular and membrane-associated responses to DEP under controlled conditions.

### 2.4. Cell Viability

The viability of A549 cells was determined by the MTT (3-[4,5-dimethylthiazol-2-yl]-2,5-diphenyl-2H-tetrazolium bromide) assay [27]. After the treatments (as described below), MTT stock solution (5 mg/mL) was added to each well to a final concentration of 1.2 mM, and the cells were incubated at 37 °C for 3 h. The functional mitochondrial succinate dehydrogenases in viable cells can convert MTT to formazan, which produces a blue color. The accumulation of formazan directly reflects mitochondrial activity as an indirect measure of cell viability. Finally, the MTT solution was removed, and the reaction was stopped by adding EtOH. After 30 min of stirring, the optical density was measured at 570 nm with 630 nm as a reference, and cell viability was normalized as a percentage of the control.

### 2.5. Preparation of Membrane-Enriched Fractions (MEF)

Cells were harvested from 12 Petri dishes (100 mm diameter) for each CTR, CTR-T, and DEP treatment to prepare the membrane-enriched fraction (MEF). The MEF, containing a fraction of endocellular membranes, was prepared according to Preti et al. [28]. Briefly, the homogenates (HOM) were centrifuged at 1000 *g* for 10 min, and after collecting the supernatants, they were resuspended in 500 μL of a solution containing 1 mM K phosphate buffer pH 7.2, 0.25 M sucrose, 0.1 mM EDTA, with a cocktail of protease inhibitors, and 1 mM PMSF. The pellets were homogenized by pipetting up and down and centrifuged three times in the same buffer, and the pooled supernatants were centrifuged at 100,000 *g* for 1 h at 4 °C. The pellet from the last centrifugation contained MEF.

The entire procedure was performed on ice, and membrane subfractionation was performed on the membrane-enriched fraction.

### 2.6. Purification of the Lipid Rafts Enriched Fraction, LRF (Lipid Rafts Fraction)

MEF obtained from CTR, CTR-T, and DEP cells was used for membrane subfractionation. The membrane pellet was resuspended in MBS containing protease inhibitors, as above, and the protein content was assayed. To maintain a constant protein/detergent ratio, an appropriate volume corresponding to 2 mg of cellular proteins was adjusted to 1 mL by mixing with MBS-containing protease inhibitors. The entire procedure was carried out on ice to preserve the integrity of the lipid rafts.

Next, an equal volume of MBS containing cold 2% Triton X-100 in MBS with protease inhibitors was added and mixed to obtain a homogeneous solution of MBS with 1% Triton. The tubes were kept on ice for 30 min. The cell lysate was centrifuged at 15,000× *g* for 30 min at 4 °C to separate the pellet containing lipid rafts (LRF, Lipid Rafts Fraction) from the supernatant containing the rest of the membrane (RoM, Rest of Membrane) [29].

### 2.7. SDS-PAGE Electrophoresis and Immunoblotting

The protein content of HOM, MEF, RoM, and LRF obtained from CTR, CTR-T, and DEP cells was analyzed by quantification with a micro-Bicinchoninic acid assay. Then, 30 µg of total protein for each sample was subjected to SDS-PAGE (10%), followed by Western blot and detection by Ponceau staining, to assess proper transfer.

Protein analysis was evaluated with specific antibodies: Ab anti-GM1 (1:300); Ab anti-Na/K-ATPase (1:500); Ab anti-ACE2 (1:500); Ab anti-ADAM17 (1:500); Ab anti-HO-1 (1:1000); Ab anti-COX2 (1:250). The appropriate horseradish peroxidase (HRP)-conjugated goat anti-rabbit or anti-mouse antibodies (1:5000) were used as secondary antibodies (1:5000).

Immunoreactive proteins were detected by Super-Signal enhanced chemiluminescence (ECL) detection kit, and semiquantitative analysis was estimated by ImageQuant™ 800 (GE Healthcare Life Sciences, Milan, Italy), 1D gel analysis program. No blinding was performed.

Staining of total protein versus a housekeeping protein more accurately represents the actual amount of loading due to less procedural and biological variation, as shown in recent studies [30,31]. Therefore, samples were normalized against the total amount of protein detected by Ponceau staining, allowing for straightforward correction for lane-to-lane variation [30,32].

### 2.8. Cytokines Analysis

Analysis of pro-inflammatory cytokines released in the medium was performed by high-sensitivity ELISA kit, provided by Enzo Life Sciences (Farmingdale, NY, USA) for human IL-6 and human IL-8, and an ELISA kit provided by AVIVA Systems Biology (San Diego, CA, USA) for sACE2, according to the manufacturer’s protocols.

### 2.9. Statistical Analysis

Data were obtained from at least three independent biological experiments, unless otherwise indicated in the figure legends. Results are presented as mean ± standard error of the mean (SEM). Pairwise comparisons among CTR, CTR-T, and DEP groups were performed using two-tailed unpaired *t*-tests, as indicated in the figure legends, with Tukey’s or Hommel’s multiple-comparison corrections applied when appropriate. Statistical significance was defined as *p* < 0.05.

For confocal microscopy analyses, Manders’ coefficient and fluorescence intensity values were calculated from multiple regions of interest/images per experimental condition, as specified in the figure legends. Image analysis was performed using standardized ImageJ macros (1.54p, Java 21.0.7); where possible, files were coded before quantification to reduce operator bias.

### 2.10. Immunofluorescence Analysis

CTR, CTR-T, and DEP cells were labeled under “non-permeabilizing” conditions with a specific antibody against ACE2 protein (GeneTex, 1:100 in complete DMEM medium) for 60 min at 4 °C and subsequently with Alexa Fluor 594-conjugated Cholerae toxin B subunit, (1:100 in complete DMEM medium) for 2 h at 4 °C with shaking. With this immunostaining only the epitopes exposed in extracellular positions are subject to antibody binding allowing to visualize only those receptors that are actually expressed on the membrane, eliminating the component undergoing synthesis or spontaneous recycling, which could interfere with subsequent analyses. To study co-localization at this stage, cholera toxin was used due to its ability to preferentially bind to GM1 and also to GM2 [33], markers of lipid rafts. In particular, specific labeling with WGA (wheat germ agglutinin), a fluorescent lectin that selectively binds N-acetylglucosamine and sialic acid residues present on the plasma membrane, was used in order to confirm the correct subcellular localization of the analyzed fluorescence).

After incubation, the cells were fixed in paraformaldehyde (PFA 4%) for 15 min at room temperature. PFA was removed by a wash with PBS-Ca^2+^/Mg^2+^, and samples were washed twice in PBS+ Ca^2+^/Mg^2+^ and stored at 4 °C for at least 16 h to remove any auto-fluorescent component. Then cells were incubated for 2 h at room temperature with Alexa Fluor™ 488-conjugated anti-rabbit secondary antibody for ACE2 primary antibody labeling (1:100 in GDB: 0.02 M sodium phosphate buffer, pH 7.4, containing 0.45 M NaCl, 0.2% (*w*/*v*) gelatin and 0.3% (*v*/*v*) Triton X-100) and counterstained with Hoechst 33,342 to visualize the nuclei. The presence of a detergent such as Triton X-100 allows permeabilization of cell membranes while nonspecific binding sites are saturated by bovine gelatin components. The excess antibody was removed with three wash in PBS+ Ca^2+^/Mg^2+^. Samples were mounted with an aqueous solution for microscopy (Fluoromount™, F4680) for subsequent observation under a confocal microscope.

### 2.11. Co-Localization Analysis

Images were acquired using a Zeiss LMm 710 confocal microscope with Airyscan (Carl-Zeiss-Strasse 22, Oberkochen, Baden-Wurttemberg, Germany) and then processed to determine the degree of co-localization between the ACE2 receptor and cholera toxin.

To avoid bias, experimental groups were anonymized and coded prior to image acquisition and quantification.

These analyses, consistent with observations from previous work [34], were performed using the ImageJ software plug-in “JACoP: Just Another Co-localization Plugin” (https://imagej.net/ij/plugins/track/jacop2.html, accessed on 12 March 2025).

The degree of co-localization is measured by the Manders coefficient [35] calculated for the receptor signal versus the cholera toxin signal (10 areas per sample analyzed). The fluorescence intensity emitted in each acquired channel (for ACE2 and for cholera toxin) was also considered. Specifically, the images were analyzed using specially designed ImageJ macros capable of calculating the mean fluorescence intensity (MIF) or its integral (ID) by normalizing it over the number of images, area, and number of cells acquired.

The data were later analyzed by one-way ANOVA statistical test with Tukey’s pos*t*-test to compare each group.

### 2.12. Lipid Composition Analysis of LRFs

The lipid component was extracted from cell samples by Bligh and Dyer procedure with chloroform, methanol (MeOH), and MilliQ water (H_2_O) in a 2:2:1.8 *v*/*v* ratio [36]. Briefly, the samples were resuspended in 500 μL of chloroform, and then 1 mL of MeOH was added. The samples were shaken for 1 min, and 500 μL of chloroform was added again. After shaking for another 1 min, 400 μL of H_2_O was added, and the samples were left on ice for 30 min. Following centrifugation at 1000 *g* for 5 min at 25 °C, the phases separation was observed: the upper phase contained polar metabolites, the interphase contained proteins, and the lower lipid-containing organic phase was collected and transferred to a new tube. The extraction was repeated as described by adding another 500 μL of chloroform. Finally, the organic phase was dried with pure nitrogen and reconstituted in 100 μL of isopropanol (ISO). Quality control (QC) samples were prepared by pooling 10 μL of each sample in a single Eppendorf tube.

### 2.13. UHPLC-MS Analysis

Samples were analyzed using a UHPLC-MS platform comprising an Agilent 1290 II liquid chromatography system coupled to a quadrupole time-of-flight mass spectrometer (Agilent 6546 LC/Q-TOF—Agilent Technologies, Palo Alto, CA, USA) [37]. Chromatographic separation of lipids was performed using a CSH ACQUITY Premier C18 column (2.1 mm × 100 mm, 1.7 μm) (Waters, Milford, MA, USA). Mobile phase A consisted of 10 mM ammonium acetate in acetonitrile/H_2_O (60/40 *v*/*v*) and 0.1% acetic acid; mobile phase B was ISO/Phase A (90/10 *v*/*v*). Samples were analyzed at a flow rate of 0.25 mL/min with the following elution gradient: 0 min 99% A, 1 min 99% A, 1.10 min 60% A, 5 min 20% A, 11 min 20% A, 12 min 1% A, 18 min 1% A, 18.10 min 60% A, 20 min 99% A. Samples were analyzed in triplicate in both positive (2 μL injection volume) and negative (5 μL injection volume). The resolution was set at 50,000 FWHM and operated in a full scan range of *m*/*z* 100–1700. QCs were used to monitor the performance of the analysis and were injected every 5 samples. At the end of the analysis, 5 QC injections were used to collect data-dependent mode (DDA) MS/MS spectra using an iterative approach.

Data acquisition (Agilent Technologies, Santa Clara, CA, USA) was used to control the Agilent 1290 II liquid chromatography and the Agilent 6546 LC/Q-TOF mass spectrometer. Five consecutive QC injections in DDA were used to acquire MS/MS data and to construct the internal library for lipids, specifically gangliosides, based on accurate mass, MS/MS fragments, isotopic pattern, and retention time, and using online databases such as HMDB [38] and METLIN [39].

The full-scan analyzed samples were then compared with our built library based on the mass formula, isotopic pattern, and retention time, and integrated using MassHunter Profinder. (Agilent Technologies, Santa Clara, CA, USA). Lipid features were evaluated based on their reproducibility in QC samples, and features showing a coefficient of variation (CV) greater than 30% across QC injections were excluded from further statistical analyses. No relevant analytical drift was observed during the run sequence; therefore, no QC-based signal correction was applied. Univariate and multivariate statistical analysis were performed using MetaboAnalyst 6.0 [40] and GraphPad Prism 9.5 (GraphPad Software, Boston, MA, USA, www.graphpad.com). Data were first normalized considering the sum of the signals and then log2 transformed to obtain a normal distribution and perform parametric test. For individual gangliosides species, *p*-values were adjusted for multiple testing using the Benjamini–Hochberg false discovery rate (FDR) procedure, and species were considered statistically different when FDR-adjusted *p*-value was <0.05. Class analyses, calculated by the sum of the corresponding ganglioside species, were evaluated using pairwise *t*-test, with statistical significance defined as *p*-value < 0.05. Ganglioside annotation was based on accurate mass, isotopic pattern, retention time, and MS/MS fragmentation when available, using an in-house library generated from QC samples and comparison with public databases. Data are reported in Table S1 together with formula, theoretical and experimental mass, delta ppm, retention time and *p*-values.

## 3. Results

### 3.1. Determination of Cell Viability by MTT Assay

DEP was chosen as a model of air pollution generated by motor vehicle traffic. A549 lung alveolar cells were treated with 1.6 µg/mL DEP [25], in a complete medium supplemented with Tween-20, for 48 h.

To verify that the treatments did not generate toxicity, cell viability was assessed by the MTT assay, which measures the mitochondrial activity directly related to cell viability.

The viability values were compared with those obtained from the control cells (CTR) and the cells treated with 0.0001% Tween-20 (CTR-T). The CTR-T condition was required because the DEP must be dissolved in the presence of Tween-20.

DEP treatment did not significantly affect viability compared with the control (CTR) or Tween-treated control (CTR-T), indicating the absence of cytotoxic effects (Figure S1). Therefore, a DEP dose of 1.6 µg/mL was used for subsequent experiments.

### 3.2. Lipid Rafts Isolation

Lipid rafts are specific membrane regions enriched in cholesterol and glycosphingolipids. These domains appear to facilitate SARS-CoV-2 entry into cells. In the early stage of infection, the receptor-binding domain (RBD) of the viral spike protein preferentially binds to sialylated glycans of GM1 and GM2 gangliosides. This interaction promotes the recognition of ACE2 by the spike protein, thus facilitating viral entry into cells [19].

A membrane-enriched fraction (MEF), obtained from A549 cells under different experimental conditions (CTR, CTR-T, and DEP), was treated with a cold non-ionic detergent to separate the lipid raft-enriched fraction (LRF) from the rest of membrane (RoM).

**Figure 1:**
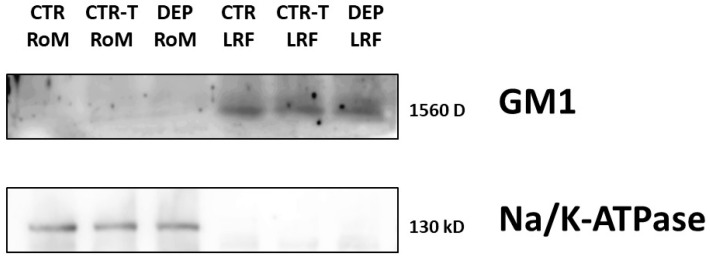
Representative immunoblotting images of GM1 and Na-K ATPase levels in the two different membrane fractions LRF and RoM, in A549 cells treated with DEP 1.6 µg/mL for 48 h. Control cells (CTR); Control cells treated with Tween-20 (CTR-T); Cells treated with DEP 1.6 µg/mL (DEP).

The presence of GM1, as a marker of lipid rafts, and Na/K-ATPase, as a marker of RoM, was assessed by immunodetection in the obtained fractions. The enrichment of GM1 in the LRF and of Na/K-ATPase in the RoM supports the separation of lipid raft-enriched and non-raft membrane fractions under our experimental conditions. The increase in these proteins in the different fractions is the necessary and sufficient condition to indicate the good purification of the fractions (Figure 1).

### 3.3. Analysis of Protein Markers

After lipid rafts isolation, we evaluated the effects of 48 h exposure to 1.6 µg/mL of DEP on the ACE2, ADAM17, COX-2, and HO-1 protein amount and their redistribution at the membrane level. Indeed, these proteins play an essential role in inflammatory processes following exposure to air pollution and are also involved in SARS-CoV-2 pathology [4,18,41,42,43].

**Figure 2:**
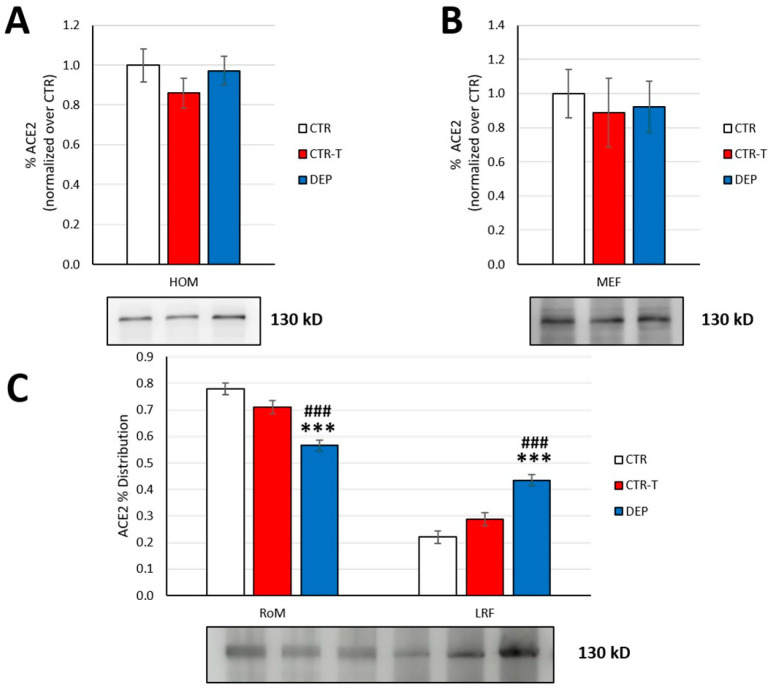
ACE2 levels in cell homogenate and membranes of A549 cells exposed to DEP 1.6 µg/mL for 48 h. Control cells (CTR); Control cells treated with Tween-20 (CTR-T); Cells treated with DEP 1.6 µg/mL (DEP). (**A**,**B**) Percentage distribution of ACE2 vs. control (CTR). (**C**) Percentage distribution of ACE2 in LRF and RoM vs. total membrane (LRF + RoM). Corresponding immunoblotting images are representative of ACE2 levels after all treatments. Proteins were normalized by the Ponceau Red value corresponding to each lane. Values are expressed as mean ± SEM (n = 3). Significance of the data was assessed by *t*-test with Hommel’s multiple-comparison correction using a threshold value α = 0.05 (RoM: DEP vs. CTR *** *p* < 0.0001, DEP vs. CTR-T ^###^ *p* = 0.0006; LRF: DEP vs. CTR *** *p* < 0.0001, DEP vs. CTR-T ^###^ *p* = 0.0006).

**ACE2**—After the treatment, no significant changes in ACE2 protein amount were observed in the HOM and in the MEF (Figure 2A,B).

ACE2 was enriched in the RoM fraction, but following DEP treatment, ACE2 levels decreased significantly in the RoM fraction compared to both CTR and CTR-T (−21.5% and −14.6%, respectively) and in parallel increased in the LRF compared to both CTR (+21.5%) and CTR-T (+14.6%) (Figure 2C).

**Figure 3:**
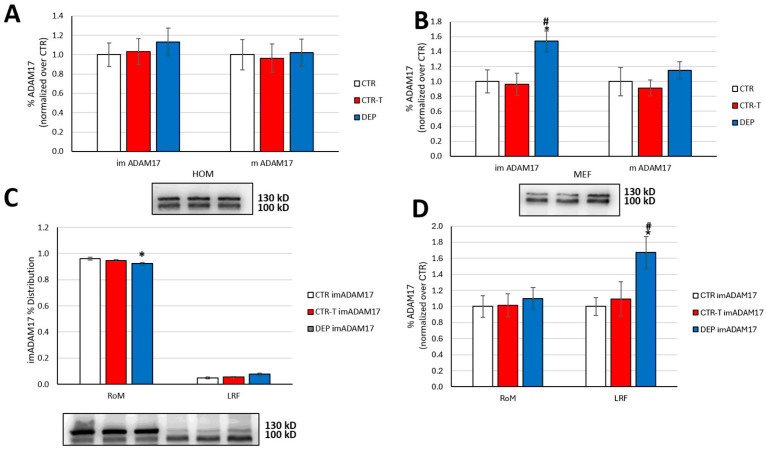
ADAM17 levels (imADAM17 and mADAM17, MW 130 KDa and 100 KDa, respectively) in cell homogenate and membranes of A549 cells exposed to DEP 1.6 µg/mL for 48 h. Control cells (CTR); Control cells treated with Tween-20 (CTR-T); Cells treated with DEP 1.6 µg/mL (DEP). (**A**,**B**,**D**) Percentage distribution of ADAM17 vs. control. (**B**) Significance of the data was assessed by *t*-test with Hommel’s multiple-comparison correction using a threshold value α = 0.05 (imADAM17 MEF: DEP vs. CTR * *p* = 0.048, DEP vs. CTR-T ^#^ *p* = 0.036). (**C**) Percentage distribution of imADAM17 in RoM and LRF vs. total membrane (RoM + LRF). Immunoblotting images are representative of ADAM17 levels after all treatments. Proteins were normalized by the Ponceau Red value corresponding to each lane. Values are expressed as mean ± SEM (n = 3). Significance of the data was assessed by *t*-test with Hommel’s multiple-comparison correction using a threshold value α = 0.05; (imADAM17 RoM: DEP vs. CTR * *p* = 0.0312). (**D**) Percentage distribution of ADAM17 vs. control Significance of the data was assessed by *t*-test with Hommel’s multiple-comparison correction using a threshold value α = 0.05; (imADAM17 LRF: DEP vs. CTR * *p* = 0.02599, DEP vs. CTR-T ^#^ *p* = 0.03466).

**ADAM17**—After the treatment, no significant changes in the mature ADAM17 (mADAM17, MW 110 KDa) or immature ADAM17 (imADAM17, MW 130 KDa) were observed in the HOM (Figure 3A).

Regarding MEF, no significant changes in mADAM17 were observed, while imADAM17 increased significantly compared to CTR and CTR-T (about +54%) (Figure 3B). Notably, imADAM17 was mainly visible in the RoM fraction where decrease significantly after DEP treatment, while it increases slightly in LRF although not significantly (Figure 3C).

Calculating the level of the protein as a percentage of the CTR revealed a significant increase in imADAM17 in lipid rafts after treatment with DEP compared to the CTR (+67.2%) and CTR-T (+57.8%) groups (Figure 3D).

**COX-2**—After DEP treatment, no significant changes in COX-2 amount were observed in either the HOM or the MEF, and it is exclusively localized in the RoM fraction (Figure S2).

**HO-1**—After DEP treatment, no significant changes in HO-1 amount were observed in either the HOM or the MEF, and it is almost exclusively localized in the RoM fraction (Figure S3).

### 3.4. Immunofluorescence Confocal Microscopy

To confirm the ACE2 data obtained by Western blotting, immunofluorescence experiments were conducted. As an initial control, the correct plasma membrane localization of ACE2 and cholera toxin B subunit was verified using wheat germ agglutinin (WGA), a fluorescent lectin that specifically recognizes N-acetylglucosamine and sialic acid residues on the cell surface.

**Figure 4:**
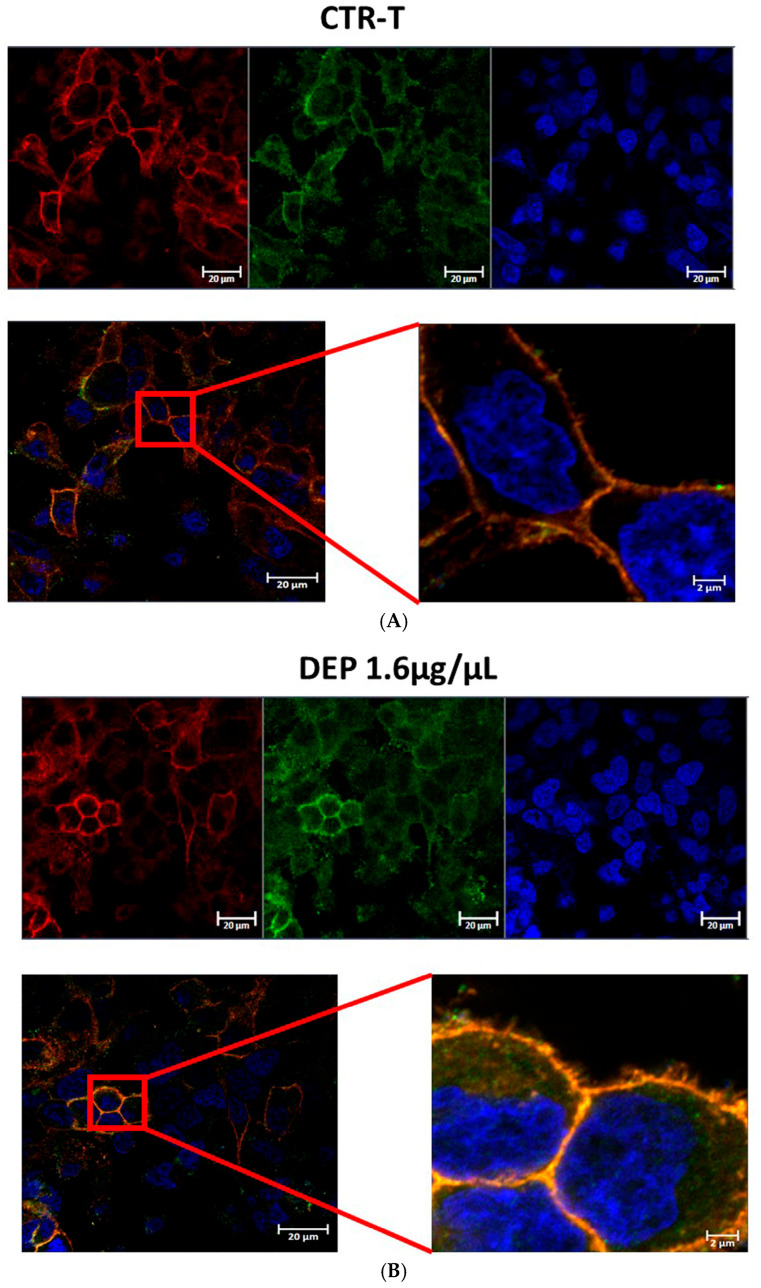
Immunofluorescence on A549 cells under control conditions (**A**) and treated with 1.6 µg/mL DEP (**B**) for 48 h. ACE2 (green) and cholera toxin subunit B (red). Co-localization at the membrane level could be appreciated by the yellow signal deriving from the merge of the two channels. In both images, the DAPI-labeled nuclei are blue. Scale bar 20 µm or 2 µm in the enlarged view.

Following treatment with 1.6 µg/mL DEP for 48 h, an increase in co-localization between ACE2 (green) and cholera toxin (red) was observed in A549 cells by immunofluorescence (Figure 4).

**Figure 5:**
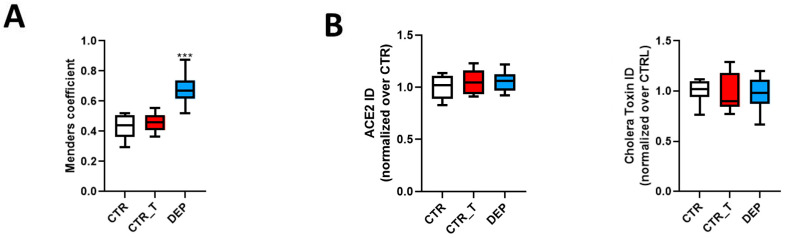
Box plot showing the colocalization of ACE2 and Cholera toxin assessed by Manders’ coefficient (**A**), 10 analysis areas per sample. Data were analyzed by *t*-test with Tukey’s pos*t*-test to compare individual groups. Significance was assessed using a threshold value α = 0.05 (DEP vs. CTR *** *p* = 0.000834; DEP vs. CTR-T *** *p* = 0.000957). Box plot showing the fluorescence intensity values (**B**) emitted by individual ACE2 and cholera toxin channels acquired separately (10 analysis areas per sample).

This increase was significant compared to both CTR (+60%) and CTR-T (+60%) (Figure 5A). Additionally, the fluorescence intensity emitted by the individual channels acquired for ACE2 and cholera toxin was measured separately. After 48 h of treatment with 1.6 µg/mL DEP, no significant changes in either ACE2 or cholera toxin were observed, confirming previous ACE2 immunoblot results (Figure 5B).

### 3.5. Evaluation of IL-6, IL-8, and sACE2 Release Following Exposure to DEP

After treating the cells with 1.6 µg/mL DEP for 48 h, we measured IL-6, IL-8, and sACE2 released into the culture medium using an ELISA assay. Indeed, it is known that PM acts on cells triggering an inflammatory state with high production of interleukins such as interleukin-6 (IL-6) and interleukin-8 (IL-8) [13]. This increase led to hypothesize that, in individuals exposed to chronic pollution, the cytokine storm following a SARS-CoV-2 infection may manifest more severely, resulting in a more complicated disease course, sometimes with fatal outcomes. Additionally, elevated levels of sACE2 in severe patients suggest that it may play an adverse role by promoting the viral spread to distant organs [44].

**Figure 6:**
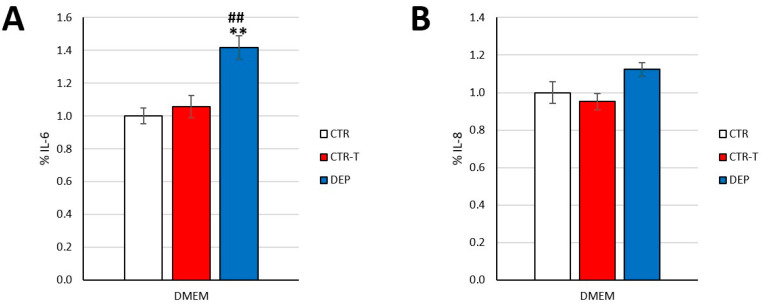
Levels of IL-6 (**A**) and IL-8 (**B**) released from A549 cells exposed to DEP 1.6 µg/mL for 48 h. Control cells (CTR); Control cells treated with Tween-20 (CTR-T); Cells treated with DEP 1.6 µg/mL (DEP). Values are calculated as a percentage of control and are expressed as mean ± SEM (n = 3). Significance of the data was assessed by *t*-test with Hommel’s multiple-comparison correction using a threshold value α = 0.05; (IL6: DEP vs. CTR ** *p* = 0.0019; DEP vs. CTR-T ^##^ *p* = 0.0039).

**IL-6-and IL-8**—Regarding pro-inflammatory cytokines, treatment induced a significant increase in IL-6 release from A549 cells compared to CTR (41.7%) and CTR-T (36%) groups (Figure 6A). However, no significant increase in IL-8 was observed (Figure 6B).

**sACE2**—After the treatment, no significant changes in sACE2 release by A549 cells were observed, though a slight, non-significant increase in sACE2 was found (Figure S4).

### 3.6. Evaluation of the Lipid Composition of Lipid Rafts

**Figure 7:**
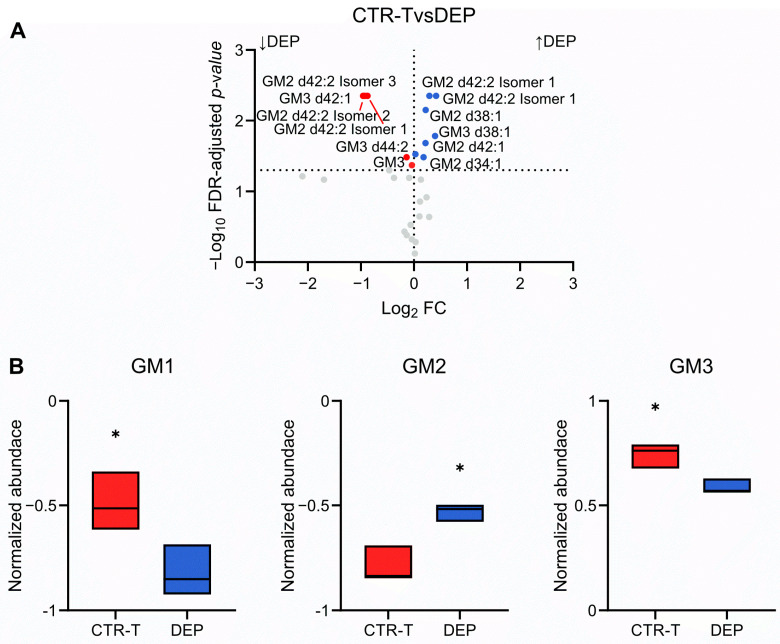
(**A**) Volcano plot showing differential abundance of gangliosides (GM1, GM2, and GM3 and their respective isomers) between CTR-T and DEP groups. Red dots indicate species down-regulated in DEP, while in blue dots the species up-regulated in DEP (*p*-value threshold 0.05, fold change 1). (**B**) Box plots showing the normalized abundance of total GM1, GM2, and GM3 gangliosides in CTR-T (red) and DEP (blue) samples. Asterisks indicate statistically significant differences between groups (*p*-value < 0.05). See Supplementary Table S1.

Lipid rafts organization is tightly regulated and evolutionarily conserved, indicating the crucial role of their lipid structure in cellular functions modulation and biological processes contribution. Therefore, after isolating lipid rafts, we examined how exposure to DEP in our experimental conditions could affect lipid rafts composition, focusing on gangliosides. The samples were analyzed using an MS-based lipidomics approach targeting specifically gangliosides. The comparison between CLTR-T and DEP samples was presented as Volcano Plot and Boxplot (Figure 7). The Volcano Plot (Figure 7A) revealed decreased levels of several GM1 and GM3 in DEP, while GM2 species were more abundant following DEP treatment. The Boxplots summarized the total abundances of GM1, GM2 and GM3. As highlighted in Figure 7B, GM1 and GM3 exhibited a significant decrease in DEP compared to CTRL-T, while GM2 showed an opposite trend, resulting significantly more abundant in DEP with respect to CTRL-T.

## 4. Discussion

During the COVID-19 pandemic, increasing epidemiological evidence suggested a potential link between air pollution and the severity of SARS-CoV-2 infection [6,7,8,9].

In particular, the most polluted regions, such as Lombardy, reported the highest incidence of severe cases and mortality, suggesting that exposure to atmospheric pollutants may exacerbate disease progression. Among these pollutants, diesel exhaust particles have attracted significant attention due to their known pro-inflammatory and oxidative effects in the respiratory system [4,45,46].

In the present study, we investigated the effects of short-term DEP exposure on A549 human lung alveolar epithelial cells, focusing on lipid rafts-associated molecular changes potentially relevant to SARS-CoV-2 susceptibility-related mechanisms. Exposure to 1.6 µg/mL DEP for 48 h did not result in detectable cytotoxicity, indicating that this condition allows the investigation of membrane-associated and inflammatory responses without compromising cell viability. Nevertheless, this model should be interpreted as a short-term sub-toxic in vitro stimulation model and not as a direct reproduction of chronic human exposure. Lipid rafts are specialized plasma membrane microdomains enriched in cholesterol and glycosphingolipids representing dynamic platforms for the compartmentalization of signaling pathways and for the spatial organization of receptors and regulatory proteins. Their involvement in viral infections has been extensively documented, as they facilitate viral entry, assembly, and release [47]. Indeed, numerous viruses exploit sialic acids linked to glycoproteins and gangliosides as receptors for host cell entry [48].

Recent structural and biochemical studies have revealed that the spike glycoprotein of SARS-CoV-2 contains a receptor-binding domain (RBD) in its N-terminal region capable of non-covalent interactions with sialylated glycans, specifically glycolipids such as GM1 or GM2 [19]. RBD refines pathogen attachment to lipid rafts and facilitates interaction with the ACE2 receptor protein, which is present in these domains [49]. This mechanism underscores the critical role of lipid rafts in viral infection. For this reason, we decided to deeply investigate their involvement in viral infection.

The renin-angiotensin system (RAS) plays a crucial role in the pathogenesis of inflammatory diseases associated with air pollution. Specifically, the ACE2/Ang(1–7)/MAS axis exerts anti-inflammatory and cytoprotective effects that counterbalance the pro-inflammatory ACE/AngII/AT1 pathway [10]. However, ACE2 also serves as the primary receptor for SARS-CoV-2, thereby linking RAS modulation to viral pathogenesis.

Wang et al. [50] demonstrated that, in obese and aged mice, ACE2 increases its proximity to lipid rafts and potentially facilitates viral entry. This mechanism closely resembles that observed in HIV infection, where lipid rafts mediate the initial interaction between the CD4 receptor and the virus [51].

The shift in ACE2 toward the lipid rafts-enriched fraction observed after DEP treatment suggests that DEP can modify the membrane microenvironment in which ACE2 is localized. Since ACE2 localization within specific membrane domains may influence receptor accessibility and organization, this redistribution may represent a molecular feature potentially relevant to viral attachment or entry [52]. However, the present data do not demonstrate increased SARS-CoV-2 binding, entry, or infection, and this hypothesis requires direct functional validation.

ACE2 plays a dual role in lung physiology and SARS-CoV-2 infection, acting both as a regulator of the renin–angiotensin system and as the primary receptor for viral entry. Therefore, its activity and localization are tightly regulated by proteolytic shedding.

ACE2 plays a dual role in lung physiology and SARS-CoV-2 infection, acting both as a regulator of the renin–angiotensin system and as the primary receptor for viral entry. Therefore, its activity, availability, and membrane localization are tightly regulated, including through proteolytic shedding. In addition to the well-characterized cleavage by the serine protease TMPRSS2, ACE2 can also be cleaved by the mem-brane metalloproteases ADAM17 and ADAM10 [53,54,55]. Both ADAM10 and ADAM17 are synthesized as inactive precursors in the endoplasmic reticulum, which contain an inhibitory pro-domain that prevents premature catalytic activity. Their maturation occurs in the Golgi apparatus through cleavage by furin-like pro-protein convertases, generating the mature forms that are subsequently trafficked to the plasma membrane, where substrate shedding may take place.

Among these enzymes, ADAM17 has been implicated in SARS-CoV-2-related cellular processes. Jocher et al. [53] demonstrated that pharmacological inhibition or genetic ablation of ADAM17 can reduce SARS-CoV-2 infection and Spike protein-mediated cell fusion in human lung epithelial cells. Moreover, ADAM17 trafficking and maturation can be regulated by iRhom2, which facilitates its transport from the endoplasmic reticulum to the Golgi and stabilizes the mature protein at the cell surface [54].

In our experimental model, the observed increase in immature ADAM17 (imADAM17) levels within lipid rafts fractions following DEP treatment may indicate an enhanced synthesis or recruitment of this protease in membrane microdomains. This phenomenon could reflect a preparatory state for rapid activation, consistent with the known dynamic regulation of ADAM17. However, iRhom2 was not measured in the present study; therefore, its possible involvement in DEP-induced ADAM17 redistribution remains speculative.

The proteolytic shedding of ACE2 by ADAM10 and ADAM17 leads to the generation of soluble ACE2 (sACE2), a catalytically active form released into the extracellular environment [56]. In our study, DEP exposure induced a slight but not statistically significant increase in sACE2 compared to control cells, suggesting that, under these conditions, DEP does not markedly affect ACE2 shedding.

In addition to ACE2-related mechanisms, other stress-responsive proteins such as cyclooxygenase-2 (COX-2) and heme oxygenase-1 (HO-1) have been associated with both air pollution exposure and COVID-19 pathology [57,58]. COX-2 is an inducible enzyme primarily responsible for prostanoid synthesis in response to inflammatory stimuli [59], while HO-1, encoded by the HMOX1 gene, exerts cytoprotective and anti-inflammatory effects under conditions of oxidative stress and injury [60]. Various stressors, including chronic exposure to pollutants, radiation, viral infections, and ischemic damage, upregulate HO-1 expression. In vivo studies have shown that acute DEP exposure increases COX-2 and HO-1 levels in mouse lung tissue [4]. However, in this in vitro model, DEP exposure did not significantly modify the expression of these proteins.

Exposure to airborne pollutants is known to induce a persistent pro-inflammatory state in the respiratory tract, characterized by the upregulation of cytokines such as interleukin-6 (IL-6) [13]. It has been hypothesized that chronic exposure to pollution may sensitize the immune system, predisposing individuals to an exaggerated inflammatory response, commonly referred to as a “cytokine storm, upon infection with SARS-CoV-2. This mechanism could contribute to the more severe and often fatal outcomes observed in specific populations, particularly older adults and patients with pre-existing comorbidities, who typically present elevated circulating levels of IL-6 [61].

In this experimental model, exposure of A549 cells to 1.6 µg/mL DEP for 48 h resulted in a significant increase in IL-6 release, indicating that DEP can directly trigger a pro-inflammatory response at the level of the alveolar epithelium.

The increase in IL-6 without a concomitant increase in IL-8, HO-1, and COX-2 can be explained by the specificity of the signaling pathways. IL-6 and IL-8 are primarily regulated by the NF-κB transcription factor and the MAPK pathway. Although both IL-6 and IL-8 bind the classic inflammatory factor NF-κB, their gene promoters differ in their interactions with other factors [62,63].

DEP are known to induce a strong pro-inflammatory and general oxidative response; the difference between IL-6 and IL-8 lies in the specific chemical components of the particulates and in the intracellular mechanisms they activate. In fact, DEP are not simply particles of elemental carbon, but carry on their surface a complex mixture of organic compounds, including polycyclic aromatic hydrocarbons (PAHs). PAHs diffuse across the membrane and selectively activate the cytosolic AhR receptor. It has been widely demonstrated that the AhR pathway has a direct, preferential mechanism for promoting IL-6 transcription, often independently of or with kinetics that are completely different from those of IL-8 [63,64].

As for HO-1, it is a cytoprotective and antioxidant protein whose expression is primarily regulated by the Nrf2 transcription factor (linked to antioxidant response elements, AREs). If the stimulus is purely inflammatory and does not induce severe oxidative stress or an accumulation of free heme, the Nrf2 pathway may remain silent, leaving HO-1 levels unchanged [62]. COX-2, although regulated by NF-κB, is strongly influenced by specific cofactors and modulators [65].

In an experimental model (in vitro or in vivo), sampling at an early stage of inflammation may reveal the presence of interleukins, particularly IL-6, whereas complex enzymes such as COX-2 or HO-1 require more time to be transcribed, translated, and accumulated in measurable quantities. Therefore, we are likely observing a pure inflammatory response, not associated with severe oxidative stress and temporally or biochemically decoupled from the eicosanoid pathway.

To corroborate the biochemical data obtained by immunoblotting, immunofluorescence analyses were performed directly on cultured A549 cells. Labeling was conducted at 4 °C under non-permeabilizing conditions, ensuring that only extracellular epitopes were accessible to antibody binding. This experimental approach allowed visualization of receptors effectively exposed on the plasma membrane, excluding intracellular ACE2 undergoing synthesis or recycling, and in this condition, we evaluated the degree of co-localization between ACE2 and cholera toxin.

The results confirmed the findings obtained from immunoblotting experiments, showing a marked increase in the co-localization of ACE2 with lipid rafts following DEP treatment, suggesting that DEP exposure may trigger reshaping of ACE2 within the plasma membrane, favoring its association with lipid rafts. Such a reorganization could increase the likelihood of interaction between ACE2 and the SARS-CoV-2 spike protein, thereby facilitating viral attachment and entry.

Finally, we investigated potential alterations in the lipid composition of lipid rafts. Lipids are fundamental constituents of cell membranes and play critical roles in numerous cellular processes, including signal transduction, membrane trafficking, and energy homeostasis. Their function is highly dependent on structural organization, and even subtle changes in lipid composition can have a profound impact on cellular physiology [66].

Lipidomic analysis by ultra-high-performance liquid chromatography coupled to high-resolution Mass Spectrometry (UHPLC-MS) revealed that DEP exposure induced a significant increase in GM2 and a concomitant decrease in GM1 and GM3 levels. Nguyen et al. [67], to examine the role of glycans in SARS-CoV-2 infection, analyzed the binding of a library of glycans to SARS-CoV-2 RBD. Blocking glycolipid biosynthesis produced similar decreases in RBD binding and viral infection, pointing to RBD-glycolipid interactions as critical for SARS-CoV-2 infection of cells. The study reveals that the receptor-binding domain (RBD) of the spike (S) protein on SARS-CoV-2 recognizes oligosaccharides-containing sialic acid (Sia), with preference for monosialylated gangliosides [67].

In immunofluorescence, no difference in the amount of cholera toxin is noted following DEP treatment. While cholera toxin B primarily targets GM1, the maintenance of this signal despite the MS-detected decrease in GM1 suggests that the increase in GM2 may preserve the overall structural integrity or total “raft area” of these domains, or that DEP induces a specific ganglioside species “flip” without reducing total rafts. These data suggest that exposure to DEP does not cause a decrease in the number of lipid rafts, but rather changes their composition.

Taken together, these data suggest that exposure to DEP may induce lipid rafts remodeling, potentially linking DEP exposure to increased susceptibility to SARS-CoV-2 infection.

## 5. Conclusions

Our data support the hypothesis that DEP exposure might increase pulmonary cell susceptibility to SARS-CoV-2 through several synergistic mechanisms: the spatial redistribution of ACE2 into lipid rafts, the targeted recruitment of ADAM17 to membrane microdomains, and the induction of a pro-inflammatory microenvironment. Moreover, the observed lipidomic remodeling, specifically the shift in ganglioside composition, suggests a biochemical “priming” of the plasma membrane that may further facilitate viral attachment and entry. Recently, Pastey et al. (2026) [68] demonstrated that lipid rafts are crucial platforms for SARS-CoV-2 and that the disruption of rafts impairs this process, highlighting cholesterol-rich membranes as essential for coronavirus assembly. However, the present data do not demonstrate increased SARS-CoV-2 binding, entry, or infection, and this hypothesis requires direct functional validation, which will be the subject of the future study.

Ultimately, identifying lipid rafts as a “preferential gateway” for SARS-CoV-2 provides a critical framework for developing novel therapeutic interventions aimed at intercepting the early stages of viral infection in populations chronically exposed to air pollution.

## Data Availability

The data presented in this study are available on request from the corresponding authors.

## Supplementary Materials

The following supporting information can be downloaded at: https://www.mdpi.com/article/10.3390/toxics14070642/s1. Table S1: Identification and statistical analysis of ganglioside species detected in DEP-exposed samples: For each detected ganglioside species, the table reports molecular formula, theoretical and experimental masses, mass accuracy (Δppm), chromatographic retention time, identification criteria (accurate mass, isotopic pattern, and MS/MS fragmentation), *p*-value, − log10 (*p*-value), and direction of change after DEP exposure. Class-level analyses for GM1, GM2, and GM3 are reported at the bottom of the table. Figure S1: Effect on cell viability of treatment with DEP 1.6 μg/mL for 48 h, in A549 cells. Values are expressed as mean ± SEM (n = 3). Significance of the data was assessed by *t*-test with Hommel’s multiple comparison correction using a threshold value α = 0.05. Control cells (CTR); control cells treated with Tween-20 (CTR-T); cells treated with DEP 1.6 μg/mL (DEP). Figure S2: COX-2 levels in cell homogenate and membranes of A549 cells exposed to DEP 1.6 μg/mL for 48 h. Control cells (CTR); Control cells treated with Tween-20 (CTR-T); Cells treated with DEP 1.6 μg/mL (DEP). Panels (A) and (B) Percentage distribution of COX-2 vs. control. Panel (C) Percentage distribution of COX-2 in RoM and LRF vs. total membrane (RoM + LRF). Immunoblotting images are representative of COX-2 levels after all treatments. Proteins were normalized by the Ponceau Red value corresponding to each lane. Values are expressed as mean ± SEM (n = 3). Significance of the data was assessed by *t*-test with Hommel’s multiple comparison correction using a threshold value α = 0.05. Figure S3: HO-1 levels in the cell homogenate and membranes of A549 cells exposed to DEP 1.6 μg/mL for 48 h. Control cells (CTR); Control cells treated with Tween-20 (CTR-T); Cells treated with DEP 1.6 μg/mL (DEP). Panels (A) and (B) Percentage distribution of HO-1 vs. control. Panel (C) Percentage distribution of HO-1 in RoM and LRF vs. total membrane (RoM + LRF). Immunoblotting images are representative of HO-1 levels after all treatments. Proteins were normalized by the Ponceau Red value corresponding to each lane. Significance of the data was assessed by *t*-test with Hommel’s multiple-comparison correction using a threshold value α = 0.05; Figure S4: Levels of ACE2s released from A549 cells exposed to DEP 1.6 μg/mL for 48 h. Control cells (CTR); Control cells treated with Tween-20 (CTR-T); Cells treated with DEP 1.6 μg/mL (DEP). Values are calculated as a percentage of control and are expressed as mean ± SEM (n = 3). Significance of the data was assessed by *t*-test with Hommel’s multiple-comparison correction using a threshold value α = 0.05.
