# Supplementary material for: Diesel Exhaust Particles Remodel Lipid Raft-Associated Molecular Features Potentially Relevant to SARS-CoV-2 Susceptibility in A549 Cells
Source: Toxics. 2026 Jul 22;14(7):642. doi: 10.3390/toxics14070642 (PMC13417371; doi:10.3390/toxics14070642)
Supplement: Supplementary file 1 [file toxics-14-00642-s001.zip › Supplementary Figures.pdf]

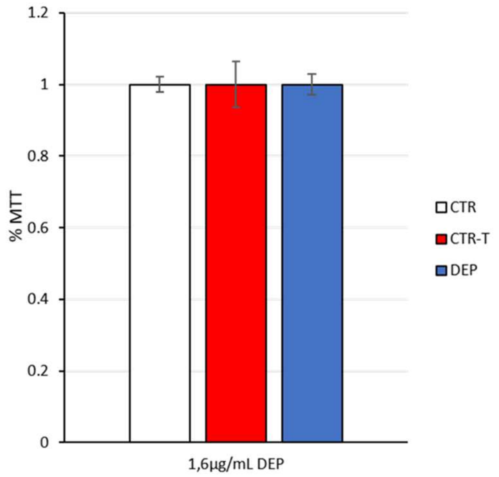

Figure S1: Effect on cell viability of treatment with DEP 1.6 µg/mL for 48 h, in A549 cells. Values are expressed as mean ± SEM (n = 3). Significance of the data was assessed by *t*-test with Hommel's multiple-comparison correction using a threshold value α = 0.05. Control cells (CTR); control cells treated with Tween-20 (CTR-T); cells treated with DEP 1.6 µg/mL (DEP).

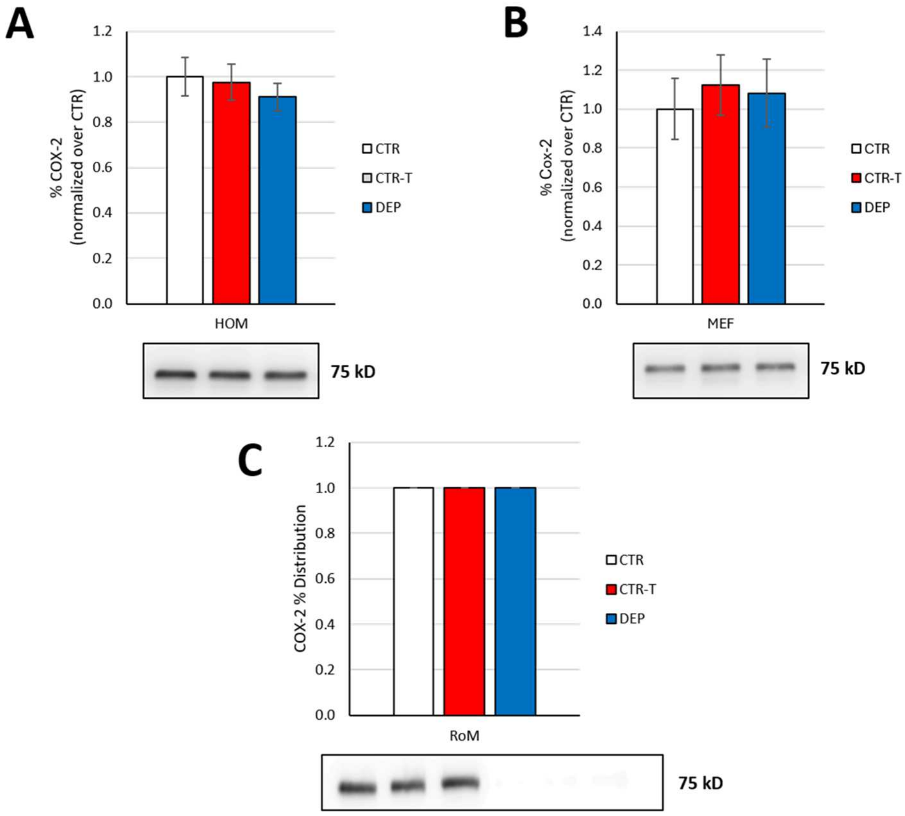

Figure S2: COX-2 levels in cell homogenate and membranes of A549 cells exposed to DEP 1.6 µg/mL for 48 h. Control cells (CTR); Control cells treated with Tween-20 (CTR-T); Cells treated with DEP 1.6 µg/mL (DEP). Panels (A) and (B) Percentage distribution of COX-2 vs. control. Panel (C) Percentage distribution of COX-2 in RoM and LRF vs. total membrane (RoM + LRF). Immunoblotting images are representative of COX-2 levels after all treatments. Proteins were normalized by the Ponceau Red value corresponding to each lane. Values are expressed as mean ± SEM (n = 3). Significance of the data was assessed by *t*-test with Hommel's multiple-comparison correction using a threshold value α = 0.05.

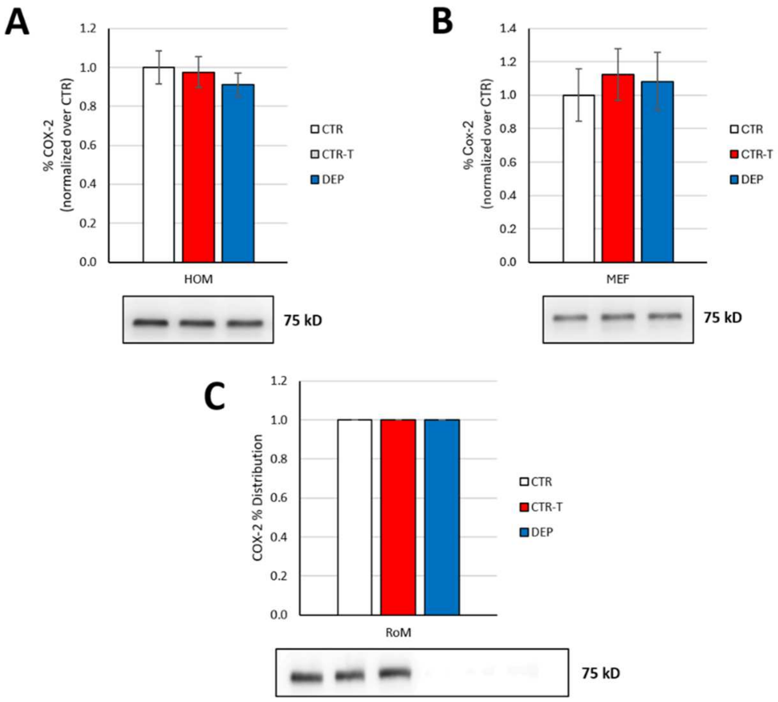

Figure S3: HO-1 levels in the cell homogenate and membranes of A549 cells exposed to DEP 1.6 µg/mL for 48 h. Control cells (CTR); Control cells treated with Tween-20 (CTR-T); Cells treated with DEP 1.6 µg/mL (DEP). Panels (A) and (B) Percentage distribution of HO-1 vs. control. Panel (C) Percentage distribution of HO-1 in RoM and LRF vs. total membrane (RoM + LRF). Immunoblotting images are representative of HO-1 levels after all treatments. Proteins were normalized by the Ponceau Red value corresponding to each lane. Significance of the data was assessed by *t*-test with Hommel's multiple-comparison correction using a threshold value α = 0.05.

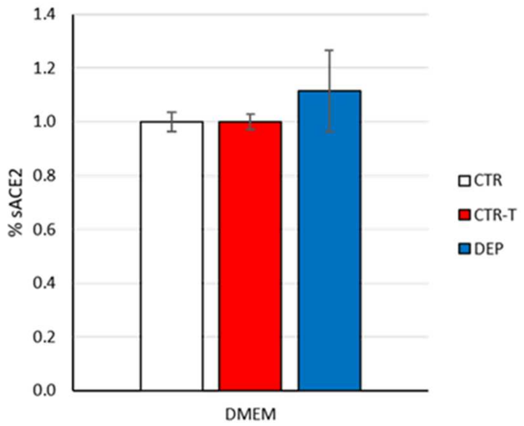

Figure S4: Levels of ACE2s released from A549 cells exposed to DEP 1.6 µg/mL for 48 h. Control cells (CTR); Control cells treated with Tween-20 (CTR-T); Cells treated with DEP 1.6 µg/mL (DEP). Values are calculated as a percentage of control and are expressed as mean ± SEM (n = 3). Significance of the data was assessed by t-test with Hommel's multiple-comparison correction using a threshold value $\alpha = 0.05$
