# Supplementary material for: Diesel Exhaust Particles Remodel Lipid Raft-Associated Molecular Features Potentially Relevant to SARS-CoV-2 Susceptibility in A549 Cells
Source: Toxics. 2026 Jul 22;14(7):642. doi: 10.3390/toxics14070642 (PMC13417371; doi:10.3390/toxics14070642)
Supplement: Supplementary file 1 [file toxics-14-00642-s001.zip › Supplementary Table S1.pdf]

Table S1: Identification and statistical analysis of ganglioside species detected in DEP-exposed samples: For each detected ganglioside species, the table reports molecular formula, theoretical and experimental masses, mass accuracy (△ppm), chromatographic retention time, identification criteria (accurate mass, isotopic pattern, and MS/MS fragmentation), p-value, − log10 (p-value), and direction of change after DEP exposure. Class-level analyses for GM1, GM2, and GM3 are reported at the bottom of the table.

Metabolite	Formula	Theoretical Mass	Experimental mass	Delta ppm	Retention Time (min ±0.3)	Identification	FDR-adjusted *p-value*	"-log10 FDR-adjusted p	Variation
asialo-GM1 d34:1	C60 H110 N2 O23	1226.7499	1226.7544	3.66822936	6.38	MS/MS fragmentation, accurate mass, isotopic pattern	0.4765	0.32194	NS
asialo-GM2 d34:1	C54 H100 N2 O18	1064.6971	1064.7042	6.668563294	6.5	MS/MS fragmentation, accurate mass, isotopic pattern	0.29707	0.52714	NS
GM1 d34:1	C71 H127 N3 O31	1517.8454	1517.8447	-0.461180038	5.42	MS/MS fragmentation, accurate mass, isotopic pattern	0.37037	0.43136	NS
GM1 d42:1	C79 H143 N3 O31	1629.9706	1629.9681	-1.533769996	6.5	MS/MS fragmentation, accurate mass, isotopic pattern	0.061147	1.2136	NS
GM1 d42:2	C79 H141 N3 O31	1627.9549	1627.953	-1.167108499	6.32	MS/MS fragmentation, accurate mass, isotopic pattern	0.068147	1.1666	NS
GM2 d34:1	C65 H117 N3 O26	1355.7925	1355.7928	0.221272798	5.43	MS/MS fragmentation, accurate mass, isotopic pattern	0.032771	1.4845	↑ DEP
GM2 d36:1	C67 H121 N3 O26	1383.8238	1383.8237	-0.072263535	5.76	MS/MS fragmentation, accurate mass, isotopic pattern	0.13893	0.8572	NS
GM2 d38:1	C69 H125 N3 O26	1411.8551	1411.855	-0.070828798	6.06	MS/MS fragmentation, accurate mass, isotopic pattern	0.007045	2.1521	↑ DEP
GM2 d40:1_isomer1	C71 H129 N3 O26	1439.8864	1439.8802	-4.305895243	6.07	MS/MS fragmentation, accurate mass, isotopic pattern	0.23038	0.63756	NS
GM2 d40:1_isomer2	C71 H129 N3 O26	1439.8864	1439.8868	0.277799693	6.32	MS/MS fragmentation, accurate mass, isotopic pattern	0.22583	0.64623	NS
GM2 d42:1	C73 H133 N3 O26	1467.9177	1467.9096	-5.518020527	6.32	MS/MS fragmentation, accurate mass, isotopic pattern	0.020702	1.684	↑ DEP
GM2 d42:2_Isomer1	C73 H131 N3 O26	1465.9021	1465.8938	-5.662042506	6.08	MS/MS fragmentation, accurate mass, isotopic pattern	0.0044481	2.3518	↑ DEP
GM2 d42:2_Isomer2	C73 H131 N3 O26	1465.9021	1465.9022	0.06821738	6.31	MS/MS fragmentation, accurate mass, isotopic pattern	0.0044481	2.3518	↑ DEP
GM2 d42:2_Isomer3	C73 H133 N3 O26	1465.9021	1465.8941	-5.457390367	6.51	MS/MS fragmentation, accurate mass, isotopic pattern	0.0044481	2.3518	↓ DEP
GM2 d44:1_Isomer1	C75 H137 N3 O26	1495.949	1495.9456	-2.272804755	6.5	MS/MS fragmentation, accurate mass, isotopic pattern	0.050381	1.2977	NS
GM2 d44:1_Isomer2	C75 H137 N3 O26	1495.949	1495.9484	-0.401083192	6.77	MS/MS fragmentation, accurate mass, isotopic pattern	0.12106	0.91698	NS
GM2 d44:2	C75 H135 N3 O26	1493.9334	1493.9331	-0.200812165	6.5	MS/MS fragmentation, accurate mass, isotopic pattern	0.064472	1.1906	NS
GM3 d34:1	C57 H104 N2 O21	1152.7132	1152.7137	0.433759239	5.46	MS/MS fragmentation, accurate mass, isotopic pattern	0.068147	1.1666	NS
GM3 d36:1	C59 H108 N2 O21	1180.7445	1180.7444	-0.084692328	5.78	MS/MS fragmentation, accurate mass, isotopic pattern	0.75546	0.12179	NS
GM3 d38:1	C61 H112 N2 O21	1208.7758	1208.7734	-1.985479855	6.09	MS/MS fragmentation, accurate mass, isotopic pattern	0.016476	1.7831	↑ DEP
GM3 d40:1	C63 H116 N2 O21	1236.8071	1236.8066	-0.404266761	6.33	MS/MS fragmentation, accurate mass, isotopic pattern	0.52001	0.28399	NS
GM3 d42:1	C65 H120 N2 O21	1264.8384	1264.8382	-0.158122967	6.52	MS/MS fragmentation, accurate mass, isotopic pattern	0.0044481	2.3518	↓ DEP
GM3 d42:2	C65 H118 N2 O21	1262.8227	1262.8225	-0.15837536	6.33	MS/MS fragmentation, accurate mass, isotopic pattern	0.032771	1.4845	↓ DEP
GM3 d44:1	C67 H124 N2 O21	1292.8697	1292.869	-0.541431205	6.79	MS/MS fragmentation, accurate mass, isotopic pattern	0.41534	0.38159	NS
GM3 d44:2	C67 H122 N2 O21	1290.854	1290.8536	-0.309872379	6.5	MS/MS fragmentation, accurate mass, isotopic pattern	0.0044481	2.3518	↓ DEP
Sum GM1							0.064472	1.1906	NS
Sum GM2							0.029813	1.5256	↑ DEP
Sum GM3							0.042431	1.3723	↓ DEP
